# Iron Overload in Gastric Mucosa: Underdiagnosed Condition Rarely Documented in Clinical and Pathology Reports

**DOI:** 10.7759/cureus.8234

**Published:** 2020-05-22

**Authors:** Monica Onorati, Marta Nicola, Anna Renda, Mauro Lancia, Franca Di Nuovo

**Affiliations:** 1 Pathology, ASST Rhodense, Garbagnate Milanese, ITA; 2 Pathology, G. Salvini Hospital, Garbagnate Milanese, ITA

**Keywords:** iron-pill gastritis, mucosal injury, oral ferrous tablets

## Abstract

The iron-deficiency anemia is a common disorder worldwide. It is widely treated with oral iron supplements as ferrous sulfate compound in pill or tablet form, and continuous therapy can induce gastric diseases. The diagnosis of this unusual drug-induced disease is based on the endoscopic findings and the histopathological biopsy examination, because the clinical symptoms are vague and non-specific. Herein we report five cases of iron pill-induced gastritis after oral ferrous sulfate administration.

The aim of this report is to underline that iron pill-induced gastritis is an under-diagnosed entity that must be kept in mind when patients undergo chronic iron-pill therapy because it can carry severe upper digestive tract complications. Moreover, we would speculate about the potential tumorigenic role of iron intake in iron-induced gastric inflammation.

## Introduction

Oral ferrous sulfate supplementation is used for prevention and treatment of iron-deficiency anemia that according to the World Health Organization afflicts approximately 25% of the population [1]. Exposure to therapeutic doses of oral ferrous sulfate induces gastric injuries, presumably by the oxidation of iron from the ferrous to the ferric form. Gastrointestinal damage frequently occurs in older patients, but it can also be encountered in children [2]. The diagnosis of this iatrogenic entity is mainly based on the endoscopic findings and on the histopathological biopsy examination. The symptoms are non-specific and can be represented by nausea, vomiting, abdominal pain or in complicated cases by upper digestive hemorrhage. Esophagogastroscopy reveals multiple brown deposits on the surface of the gastric mucosa associated with erosions and non-bleeding gastric ulcer. Mucosal biopsies show a peculiar feature represented by excessive iron deposition in macrophages, stroma, glandular epithelium and in the blood small vessels associated with severe injuries such as gastric erosions, gastric ulceration, and mucosal necrosis [3]. The purpose of this report is to document the unusual clinical and histomorphological features of iron-related injury in gastric biopsy specimens of five patients treated with chronic oral iron tablets at therapeutic dose. Finally, we would like to argue about the role of iron intake in iron-induced gastric inflammation as potential trigger in gastric mucosa tumorigenesis.

## Case presentation

**Figure 1:**
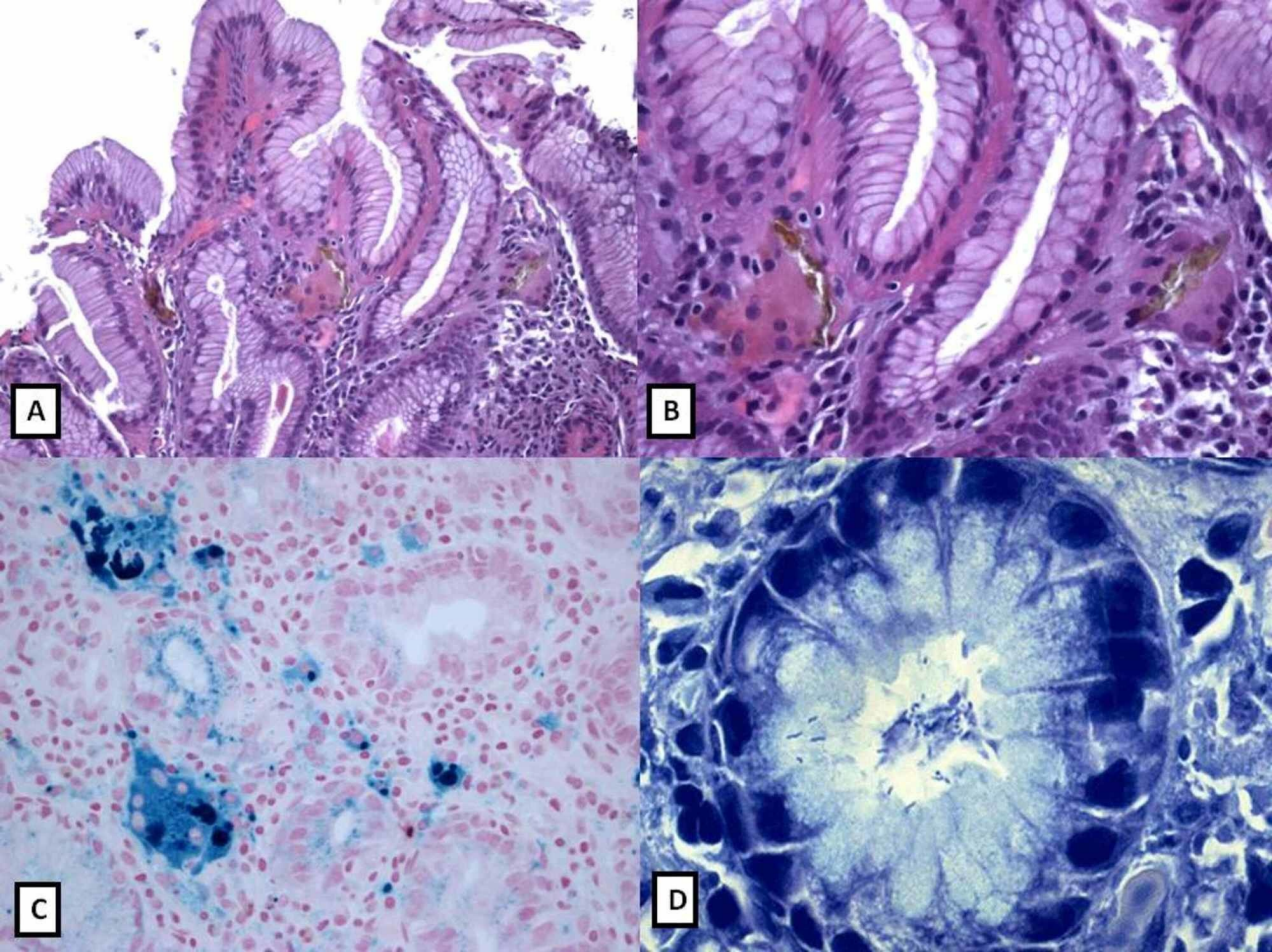
Histology of iron overload in gastric mucosa of patient number one as mentioned in Table 1 A, B: Gastric bioptic specimens showing stroma cells and epithelium iron deposition (H&E: 10x; 40x). C: Perl's stain showing a bluish coloration of iron deposits (40x). D: Giemsa stain showing numerous Helicobacter pylori curved bacteria in foveolar epithelium of the gastric mucosa (60x).

Between January 2015 and December 2019, five patients, three men and two women (ranging from 55 to 88 years; median age: 77 years), presented to our attention for vague epigastric pain associated with a persistent iron deficiency anemia, fatigue and weight loss. They suffered from different clinical conditions varying from diabetes to chronic arthritis, cardiovascular diseases, hepatic cirrhosis and colorectal cancer. They had no past medical history of blood transfusions but they were taking oral iron tablets at therapeutic doses for their anemia (Table 1). Clinical data related to blood analysis showed normal serum ferritin level, so the diagnoses of hemochromatosis and hemosiderosis were ruled out. Due to the persistent anemia, the patients underwent a diagnostic upper digestive endoscopy that showed different gastric endoscopic panels, ranging from brownish and bluish discoloration mucosal patches, surrounded by mild haemorrhage, to multiple gastric erosions and gastric mucosa ulcers. Multiple biopsies were taken from fundic, corpus and antral mucosa of each patient. The biological specimens were sent to pathology unit and fixed in 10% buffered formaldehyde. Then they were routinely processed and stained with hematoxylin-eosin (H&E). Other sections were cut and used for ancillary studies such as Perl's and Giemsa stains. On microscopic examination, at a lower magnification, histological staining for H&E showed the gastric mucosa with superficial oedema, chronic inflammation and a layer of brownish granular pigment. It covered the epithelial surface and extended into the superficial gastric pits and within the macrophages in the lamina propria. Some of the pigment appeared crystalline in a non-polarizing, refractile and coarsely fibrillar way, in contrast to hemosiderin, that was finely granular and intracellular. The bluish pigment was present in an extracellular location surrounded by scattered multinucleated giant cells. Perl's stain was performed showing plurifocal positivity for iron deposition that gave a diffuse bluish tint to the macrophage cytoplasm and in the lamina propria. Moreover, two of the patients showed curved Helicobacter pylori organisms, highlighted on special stain Giemsa, floating in the mucous on the foveolar surface as well as within the crypts (Figure 1). In consideration of the clinical data and of the morphological findings, the final histopathological diagnosis was: iron pill chronic gastritis. Patients underwent oral iron tablets suspension despite their persistent anemia and a clinical improvement was observed. After one month, upper gastrointestinal endoscopy showed normal appearing gastric mucosa. Repeated endoscopic biopsy did not show iron deposition either within the epithelial cells or within the lamina propria.

**Table 1:** Clinical and histological findings of our five cases

Patient	Age	Sex	Oral iron therapy	Site of biopsy	Clinical condition	Histological pattern of iron distribution
1	85	Female	Yes	Gastric fundus	Diabetes	Deposition in stromal and epithelial cells
2	81	Female	Yes	Gastric fundus	Chronic arthritis	Extracellular deposition
3	88	Male	Yes	Gastric antrum	Hepatic cirrhosis	Extracellular deposition
4	55	Male	Yes	Gastric antrum	Colorectal cancer	Deposition in stromal and epithelial cells
5	85	Male	Yes	Gastric antrum	Cardiovascular disease	Extracellular deposition

## Discussion

Iron pill supplementation-induced gastric injury is probably fairly common but an under-recognized entity. It affects patients with iron deficiency anemia treated with ferrous sulfate supplementation in pill or tablet form, likely because of a concentration effect, but it has not been noted to occur in patients who take a liquid form of iron supplementation. Endoscopically, iron pill-induced gastritis manifests as erosions and ulcerations. Iron produces a focal erosive mucosal injury similar to that caused by a chemical burn. It is supposed that iron erodes the mucosa through a direct corrosive effect that subsequently induces a local injury to the mucosa in a concentration-dependent manner, presumably by the oxidation of iron from the ferrous to the ferric form [4].

Some patients, particularly those with other comorbidities such as hemochromatosis, gastric antral vascular ectasia, and gastric adenocarcinoma among others, may have increased their susceptibility to this condition, even if significant investigation into this subject has not been undertaken. This injury often manifests as non-specific histological changes so it is very difficult to make a definitive diagnosis that can become challenging. In patients with iron pill-induced gastritis, the typical macroscopic aspect consists in erosions, erythema and yellowish-brown discoloration of the mucosa, whereas the histopathological exam reveals a degree of brown-black crystalline iron deposition, resembling hemosiderin in the epithelium, in lamina propria, in glandular lumen, even in micro-thrombi or in giant cell reaction. The crystalline iron is non-polarizing but refractile and coarsely fibrillar, in contrast to hemosiderin that is finely granular and intracellular [4]. Both are positive to Perl's iron stain.

Pathological changes, due to iron injury, have been at first classified into four patterns by several previous series (luminal pattern, lamina propria pattern, epithelial pattern and reticuloendothelial pattern) [5]. Marginean et al. and Melit et al. reclassified them subsequently into three main patterns: in type A iron deposition affects mainly macrophages, stroma, and epithelium and it is related to gastric phlogosis, ulceration and hemorrhages; type B shows iron depositions mainly at extracellular location, comprising small deposits in blood vessels, macrophages, and epithelium. Type A also called “non-specific gastric siderosis” and type B defined “iron pill gastritis” typically occur in patients receiving oral ferrous sulfate tablets. In type C pattern, deposits are present within cytoplasm of antral and fundic epithelium in patients affected by systemic iron overload [3, 6]. In our patients, at histopathological examination, we found similar multiple erosions and yellowish-brown iron deposits on the gastric mucosa mainly in lamina propria, in the glandular lumen and within foreign body giant cells, as in the patterns “type A and type B”.

Clinical data, including medical drug history, predisposing condition such as diet rich in red meat, endoscopic findings and specific histological patterns should alert the pathologists to make an accurate differential diagnosis list. Moreover, a correct diagnosis is fundamental because several epidemiological studies underline that iron pill therapy and high intake of red meat could be related to increased gastrointestinal cancer risk. These studies were conducted on a mouse model of inflammation-associated colorectal cancer. The hypothesis was that dietary iron and colonic mucosa inflammation synergically activated colonic interleukins promoting tumorigenesis [7-8]. They showed that iron staining was not detectable in the neoplastic cells suggesting that the increased iron taken by tumors did not accumulate but was instead utilized for cell proliferation. They hypothesized that specifically oral iron therapy may be detrimental in inflammatory bowel disease since it may exacerbate colonic inflammation and increase colorectal cancer risk.

The gold standard would be that the use of oral iron for the treatment of anemia should be avoided as it is unlikely to improve anemia and may exacerbate colonic inflammation increasing the risk of colorectal cancer in patients affected by chronic inflammatory bowel diseases. Also in gastric mucosa the iron has been detected in the interstitial space or in the macrophages but not in the cytoplasm of the epithelial gastric cells suggesting that it could be a factor inducing gastritis with the same mechanism hypothesized for colonic mucosa and could synergistically activate gastric interleukins promoting tumorigenesis. In contrast, other studies have demonstrated that a liquid form of iron supplement is less damaging for the gastric mucosa than solid forms. In fact the liquid form of iron does not concentrate within gastrointestinal mucosa: animal experiments demonstrated the absence of signs of mucosal damage even if lethal ferrous serum level was detected [9-10].

## Conclusions

In consideration of histological aspects observed in our patients we believe that it is important to document these lesions in pathology reports to give a valid feedback to the clinicians in order to apply a correct follow-up and a better therapy, since liquid iron has been suggested as a valid and promising alternative to the current treatment. Finally, we hypothesize that iron intake in iron-induced gastric inflammation could play a potential tumorigenic role but further studies and reports will be necessary to validate these speculative hypotheses.
